# Bibliographical

**Published:** 1880-06

**Authors:** 


### BIBLIOGRAPHICAL.

### A TREATISE ON ORAL DEFORMITIES AS A BRANCH OF MECHANICAL SURGERY.

*By Norman W. Kingsley, M. D. S., D. D. S.*

WITH OVER 350 ILLUSTRATIONS.

To those of the dental profession who have been familiar with the writings of Dr. Kingsley for the past fifteen years, (and who has not) the issue of this volume is not a surprise. Every progressive man in our profession has for years past been interested and profited by these writings.

There is embodied in this volume the results of a larger personal experience than is to be found in any other single volume, so far as I am aware. These results have been attained under the modifying influence of the advanced knowledge of principles that characterize the present time, together with an untiring perseverence, industry and devotion, to this specialty. All the abnormal conditions and irregularities of the oral cavity, particularly in respect to size and form, whether of congenital or accidental kind, are fully discussed, both in respect to the principle involved and the treatment indicated.

Not only is the large and extensive practical experience of the author embodied here, but the experience of others has been consulted and made to add, so far as practicable to the value of the work. Dr. Kingsley is thoroughly versed in the literature of the subject, and whatever is there found of value has not been overlooked. The physiology, pathology and etiology is fully and clearly considered. Almost every phase of irregularity and malformation has received attention.

The highest type of art, in two respects, has been embraced in what seems to have been the great life work of Dr. Kingsley, viz., restoration and improvement of that distinctive human faculty, speech, and the restoration to symmetry and beauty of the marred and distorted human face. The demand and importance of attention in both these directions is but slightly comprehended or understood by any who have not made the subject one of careful study.

While this work in which Dr. Kingsley stands so prominent is one largely of prosthetic character, yet it embraces much of true surgical science and art, indeed no one could attain to any desirable degree of success without a large measure of knowledge on this subject.

I do not here attempt a review of this work, for two or three reasons. Most of those who would be interested in a review have already, from reading Dr. K., a better idea of the work than could be given in an ordinary review space; and those who have not read his writings should now begin and study this book thoroughly.

The work will be a hand-book for the practitioner, a text book for the student, (it is already introduced as a text book in some of the dental colleges, and doubtless soon will be in all,) and a work of interest and value to the physician and surgeon. The style is of such a character as to please, interest and instruct the non-professional reader.

It should at once find a place in the library of every dentist in the country.

It is published by D. Appleton & Co., New York, and is for sale by Robt. Clark & Co., of this city. Price, $5.

